# Reduction of Cell Fusion by Deletion in the Hypervariable Region of the Spike Protein of Mouse Hepatitis Virus

**DOI:** 10.3390/v14020398

**Published:** 2022-02-15

**Authors:** Nipuna Tennakoon, Jihoon Ryu, Makoto Ujike, Fumihiro Taguchi, Hyun-Jin Shin

**Affiliations:** 1 College of Veterinary Medicine, Chungnam National University, Daejeon 13434, Korea; nipunatennakoon55@gmail.com; 2 Research Institute of Veterinary Medicine, Chungnam National University, Daejeon 13434, Korea; jihoon0511@cnu.ac.kr; 3 Faculty of Veterinary Medicine, Nippon Veterinary and Life Science University, Musashino, Tokyo 186-8602, Japan; ujike@nvlu.ac.jp

**Keywords:** mouse hepatitis virus, reduction, cell fusion, hypervariable region, deletion

## Abstract

Deletions in the spike gene of mouse hepatitis virus (MHV) produce several variants with diverse biological characteristics, highlighting the significance of the spike gene in viral pathogenesis. In this study, we characterized the JHM-X strain, which has a deletion in the hypervariable region (HVR) of the spike gene, compared with the cl-2 strain, which has a full spike gene. Cytopathic effects (CPEs) induced by the two strains revealed that the size of the CPE produced by cl-2 is much greater than that produced by JHM-X in delayed brain tumor (DBT) cells. Thus, this finding explains the greater fusion activity of cl-2 than JHM-X in cultured cells, and we speculate that the deletion region of the spike protein is involved in the fusion activity differences. In contrast with the fusion activity, a comparison of the virus growth kinetics revealed that the titer of JHM-X was approximately 100 times higher than that of cl-2. We found that the deletion region of the spike protein was involved in fusion activity differences, whereas cl-2 produced significantly higher luciferase activity than JHM-X upon similar expression levels of the spike protein. However, the reason behind the growth difference is still unknown. Overall, we discovered that deletion in the HVR of the spike gene could be involved in the fusion activity differences between the two strains.

## 1. Introduction

Coronaviruses belong to the family *Cornaviridae* in the order *Nidovirales,* and the term Corona reflects the crown-like spikes on the outer surface of the virus. Hence, these viruses are called coronaviruses [1]. These viruses are enveloped, positive single-stranded RNA viruses associated with a variety of acute and chronic diseases of the neurological, gastrointestinal and respiratory systems in both animals and humans [2,3,4]. Mouse hepatitis virus (MHV) causes acute, subacute, and chronic infections of the CNS in mice and rats, with symptoms ranging from fatal encephalitis to chronic demyelinating disease [5,6]. The MHV virion has a genome of approximately 32 kb packaged in a nucleocapsid (N) surrounded by three structural proteins: the membrane (M), small membrane (sM), envelope (E), and spike (S) proteins, while some strains have an optional protein called hemagglutinin esterase on the envelope [6,7,8]. Infection of the rodent central nervous system by the murine coronavirus strain JHM (MHV-JHM) causes acute and chronic neurological disease.

Spike glycoprotein is the critical component of the virion that binds to the host cell receptor and fuses with susceptible cells and subsequently fuses to virus-infected cells during viral infection [6]. The spike glycoprotein is established on the surface of the virion as a projection or peplomer and forms two subunits, S1 (N-terminal) and S2 (C-terminal), after being proteolytically processed [6,8]. It is speculated that the globular head of the spike is formed by the S1 subunit and that the S2 subunit forms the membrane-bound stalk [9]. The S1 subunit is involved in binding with the cellular receptor, while the S2 subunit is associated with subsequent membrane fusion. The spike protein expressed on the host cell membrane can also form fusions between cells [10,11].

Among the structural proteins of coronaviruses, the spike protein is involved in mediating virus entry, determining the viral host range, tissue tropism and inducing host immune responses [12,13]. The preeminent role of the S protein in viral infection indicates the importance of using it as a potential target for vaccine development and antibody-blocking therapies [14]. Previous studies have shown that monoclonal antibodies against the specific antigenic determinants on the spike protein can recognize different virulent strains, suggesting that the MHV spike protein plays a major role in its pathogenesis [15,16]. MHV spike proteins are an influential target for the initiation of both humoral and cellular immune responses at the time of in vivo infection [11,17]. Several studies have shown that spike deletion variants are naturally produced during the persistence of MHV. Generally, those variants have impressive pathogenic properties, such as reduced neurovirulence or increased tropism for white matter [6]. Those deletions in the spike gene are aligned within the HVR of the S1 subunit. The specific function of the HVR in viral pathogenesis seems to be complex and multifunctional. Moreover, deletions or mutations in the HVR are involved in the alteration of viral fusion, elimination of neutralizing antibodies and abrogation of CD8+ T-cell epitopes [11,15,16]. Previous studies on deletion variants have shown that each variant is sheltered in the central nervous system of infected mice during persistent infection, and those mutants are mostly abandoned in the spinal cord RNA of mice, causing severe neurological impairment [6], emphasizing the importance of studying MHV deletion variants.

The MHV variant cl-2 isolated from rat brains [18] has a larger S protein, while MHV JHM-X, which is a derivative of wild-type JHMV isolated in Japan [19], consists of a 153 amino acid deletion in the HVR. The biological significance of this deleted region has not been confirmed. In this study, we compare biological features between cl-2 and JHM-X viruses to attempt to understand the importance of this 153 amino acid region, which is found in cl-2 but not in JHM-X.

## 2. Materials and Methods

### 2.1. Cells and Viruses

DBT cells, a mouse astrocytoma cell line [20], were grown in Dulbecco’s modified Eagle’s medium (DMEM) (Biowest, USA) supplemented with 10% fetal bovine serum (FBS), 10% tryptose phosphate broth (TPB) and 1% antibiotic-antimycotic (AA) (Biowest, Riverside, MO, USA) and used for virus propagation and all plaque assays. Human embryonic kidney (HEK) 293T cells were maintained in DMEM containing 10% FBS, 1% AA and 1% HEPES.

MHV JHM-X [21] and cl-2 [22] virus strains were propagated, and plaques were assayed using DBT cells according to a previously described method with some modifications [23]. Viruses were inoculated into DBT cells and allowed to adsorb in a CO_2_ incubator at 37 °C for 1 h. After 1 h, viruses were removed, and the cells were cultured in maintenance medium (DMEM supplemented with 10% TPB) for 14 h. The supernatant was recovered, and cell debris was removed by centrifugation at 2000 rpm for 10 min. The separated supernatant was aliquoted into small volumes and stored at −80 °C. Stored virus titers of MHV JHM-X and MHV cl-2 were quantified and were 4 × 10^6^ and 1 × 10^5^ plaque-forming units (PFU) per 0.5 mL, respectively. Although deletion in the HVR of the JHM-X spike protein was reported previously [6], we isolated viral RNA from both virus strains to generate cDNA using TOPscript RT DryMIX (Enzynomics, Daejeon, Korea) following alignment of the amplified spike gene sequences for further confirmation of the deleted region in the HVR of the JHM-X spike protein.

### 2.2. RT-qPCR for Virus Growth Comparison

For further confirmation of the virus growth difference between two viruses in DBT cells, RT-qPCR analysis was performed using the virus infected supernatant at lower MOI. In detail, DBT cells were seeded in 24 well cell culture plates. When the cells get confluence, viruses (50 µL) were infected at the MOI of 0.0002 following incubation at 37 °C in the CO2 incubator with intermittent inverting to spread the virus equally. Followed by 1 h incubation, the virus was aspirated and the cells were washed two times with pre-warmed (37 °C) minimum essential medium (MEM) to remove the excess virus and DMEM, 10% TPB medium was added into each well. Supernatants from the virus infected wells were harvested at 6, 12, 18, 24 h post infection and stored at −80 °C. Viral RNA was isolated from 300 µL of thawed supernatant, subsequently, cDNA was prepared using the same kit components mentioned before.

**Figure 1:**
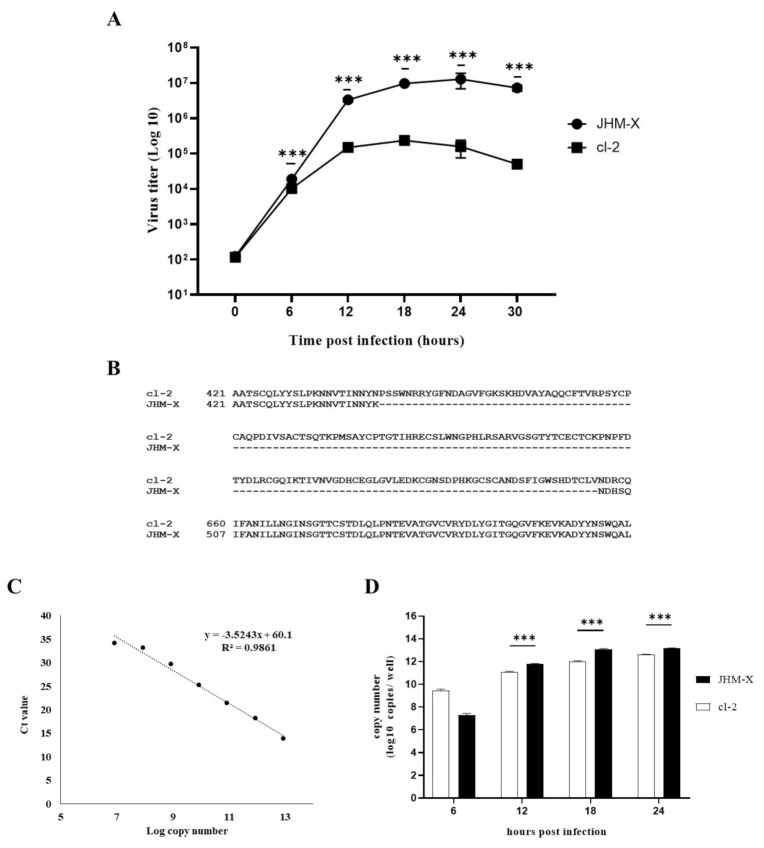
Comparison of MHV growth in DBT cells using plaque assay and RT-qPCR analysis following differences in the spike protein between the two MHV strains are indicated. (**A**) DBT cells prepared in 24-well plates were infected with either MHV JHM-X or cl-2 viruses at an MOI of 0.01, and the viruses were harvested every 6 h post inoculation. The titers (PFU/0.5 mL) were determined in cultured DBT cells. (**B**) Amino acid comparison of the region near the deletion in JHM-X with the complete JHM cl2 spike protein. (**C**) Standard curve was obtained by using the Ct values and the log copy numbers of the 10 fold serial dilutions of the MHV membrane plasmid. (**D**) DBT cells were infected with either JHM-X or cl-2 at MOI of 0.0002 following viral RNA being isolated from the harvested supernatant at each time point and cDNA was prepared. Quantitative PCR was performed to obtain the Ct values and respective copy numbers were obtained using the equation obtained from the standard curve followed by graphed against the time. *** denotes a statistically significant difference (*p* < 0.0001 determined by a Student’s *t*-test).

To quantify the cDNA copies, a standard serial 10 fold dilutions of a known amount of MHV membrane plasmid DNA (pCAGGS MHV M) was prepared. Copy numbers of standards were retained in the range between 8.448 × 10^12^ and 8.448 × 10^6^. Finally, 1 µL of cDNA from samples or serial dilutions of standards were used for the RT-qPCR analysis in a 10 µL reaction mixture containing 10 mM of each forward and reverse primers specific for MHV membrane gene (5′ GGAACTTCTCGTTGGGCATTATACT 3′ and 5′ ACCACAAGATTATCATTTTCACAACATA 3′, respectively), 5 µL of THUNDERBIRD^®^ SYBR^®^ qPCR mix (TaKaRa^®^, Kusatsu, Japan) and 2 µL of distilled water. RT-qPCR was performed in the Thermal Cycler Dice Real Time System (TaKaRa^®^, Kusatsu, Japan). A standard two step thermal cycling profile was conducted at 60 °C and finally, a single dissociation step was performed to determine the primer specificity. The standard curve was generated using the copy numbers of 10-fold serial dilutions and the respective threshold cycle (Ct) values obtained by qPCR (Figure 1C). Using the equation derived from the standard curve (y = −3.5243x + 60.1), the copy number of each sample was calculated and graphed against the time post infection (Figure 1D).

### 2.3. Syncytium (Plaque) Size and Quantification of Nucleus Number Inside a Plaque

DBT cells were prepared in 24-well cell culture plates (SPL, Pocheon-si, Korea) and infected with JHM-X or cl-2 viruses at an MOI of 0.01, which were allowed to adsorb for 1 h in a CO_2_ incubator. Viruses were aspirated, and infected cells were overlaid with DMEM containing 2% FBS and 1% methylcellulose for 15–16 h. The cells were fixed with 10% formaldehyde and then stained with 0.05% crystal violet. Following plaque observation under a microscope (Olympus IX51), the plaque area was analyzed with microscopic imaging software (eXpo ver. 5.0.1) (Figure 1B). Furthermore, DBT cells prepared in 24-well plates were infected with either MHV JHM-X or cl-2 viruses at an MOI of 0.01. Consecutively, the number of nuclei inside a syncytium was counted 9 h post infection, observed under a microscope and statistically compared using a Student’s *t*-test (Figure 1C).

### 2.4. Plasmid Construction

Construction of novel plasmids containing MHV spike proteins of cl-2 and JHM-X was performed using pTARGET cl-2 S as previously described [24]. The spike gene of MHV cl-2 was amplified using the forward primer 5′-TACCCGGGCATGCTCGAGATGCTGTTCGTCTTTATTTTACTATT-3′ and the reverse primer 5′-GGATCCTCATGGGCTGAAATATTATA-3′; thereafter, the PCR product and the pCAGGS vector were digested with the enzymes *Xho I* and *Bgl II*, and the cl-2 spike protein sequence was inserted downstream of the CMV-IE promoter, generating pCAGGS cl-2 S. The JHM-X spike protein, with a deletion of 459 bp in the 3′ region of S1, was reported previously [6]. The pCAGGS JHM-X spike protein with a 459 nucleotide deletion was constructed by removing the quoted region from the pCAGGS cl-2 S vector by performing a specific PCR in 30 µL of reaction mixture containing 2X TOPsimple™ DyeMIX–Tenuto (Enzynomics, Korea) and the specific primers presented in the table. The upstream region was amplified using a forward primer (F1) with an existing restriction enzyme site (*Xho I*) and a reverse primer (R1) with an overhang region overlapping with the downstream region next to the deleted region. Correspondingly, the downstream region was amplified using a forward primer (F2) with an overhang region overlapping with the upstream region above the deleted region and a reverse primer (R2) with an existing restriction enzyme site (*Nru I*) in the plasmid. Purified PCR products from both reactions were used as the template for the second PCR amplification with the forward primer (F1) 5′ TACCCGGGCATGCTCGAGATGCTGTTCGTCTTTATTTTACTATT 3′ and reverse primer (R2) 5′ TCGCGAACTTCTTGACCACCAGTGCAATTG 3′ in a 30 µL reaction mixture according to the manufacturer’s protocol (GeneAll^®^ Expin^TM^, GeneAll, Korea). Primer details are presented in Table 1.

### 2.5. Transfection and Virus Infection

Confluent monolayers of DBT cells plated in 6-well cell culture plates (SPL, Korea) were infected with MHV JHM-X or MHV cl-2 viruses (MOI of 0.01) in 200 µL of serum-free minimum essential medium (MEM) (Welgene, Gyeongsan-si, Korea). After one hour of incubation in a CO_2_ incubator, the virus mixture was aspirated, and the cells were cultured in antibiotic-containing DMEM supplemented with 2% FBS and 2% TPB. For the expression of recombinant MHV spike proteins, a monolayer of DBT cells was transfected with pCAGGS cl-2 S and pCAGGS JHM-X S plasmids using Lipofectamine^TM^ 3000 (Invitrogen, USA) reagent according to the manufacturer’s instructions.

### 2.6. Western Blotting

At the optimum time of plaque formation (16 h after infection or 24 h after transfection), cells were washed once with phosphate-buffered saline (PBS). A single cell suspension was prepared using 0.25% trypsin-EDTA (Gibco, USA), and the trypsin digestion was inactivated using culture medium. The cells were washed with PBS and lysed with OttimoLyse I lysis buffer (JUBIOTECH, Korea) containing protease inhibitor cocktail in Eppendorf tubes. The lysed samples were centrifuged at 15,000× *g* for 30 min at 4 °C, and the supernatant was recovered. The cell lysate was boiled in 20 µL of loading mixture containing the sample buffer (50 mM Tris HCl [pH 6.8], 5% 2-mercaptoethanol, 8% SDS, 6% glycerol, 0.001% bromophenol blue), and prepared lysates under reducing conditions (samples were boiled at 95 °C for 10 min following incubation in ice for 5 min and centrifugation at 15,000× *g* for 3 min) were separated via SDS–PAGE and the proteins were subsequently transferred to PVDF membranes. The transferred S proteins were incubated successively with anti-JHMV S2 monoclonal antibody 10 G [25] as the primary antibody, kindly provided by F. Taguchi, and then with HRP-conjugated anti-mouse IgG as the secondary antibody (CSB-PA644737, Cusabio, Houston, TX, USA). Detection was performed using Super Signal western blot Enhancer (Thermo Fisher Scientific, Waltham, MA, USA).

### 2.7. Immunofluorescence Assay

Monolayers of DBT cells grown on coverslips in a 12-well culture plate (SPL, Korea) were infected with MHV cl-2 or JHM-X viruses (MOI of 0.01) or transfected with 1.5 µg of pCAGGS cl-2 S or pCAGGS JHM-X S. Fifteen to 20 h after transfection, cells were fixed to coverslips with 4% paraformaldehyde (Biosesang, Korea) for 15 min and washed with 1X phosphate-buffered saline (PBS) (Biosesang, Korea) three times. Then, the cells were permeabilized by exposure to 1% Triton X-100 (BioShop, Korea) in PBS (PBSt) for 15 min, and the nonspecific binding sites were blocked with 2% bovine serum albumin (BSA) in PBS for 1 h. Previously reported mouse monoclonal antibodies against MHV spike protein [26] were added to cells at a 1:100 dilution, and Alexa Fluor 488-conjugated goat anti-mouse secondary antibody (#A-11001, Invitrogen, Waltham, MA, USA) diluted at 1:200 was used for detection. Finally, the nuclei were stained with Hoechst 33,342 solution (Thermo Fisher Scientific, USA) diluted at 1:10,000 in PBS, and coverslips were mounted on slides using Fluoroshield mounting medium (ab104135, Abcam, Waltham, MA, USA) for observation under a fluorescence microscope.

### 2.8. Enzyme-Linked Immunosorbent Assay

Ten micrograms of whole-cell lysates of DBT cells infected with cl-2 or JHM-X at an MOI of 0.002 or mock infected were coated on microtiter plates (SPL, Korea) at 4 °C overnight. Then, the plates coated with lysates were incubated with the monoclonal antibody 10 G diluted at 1:5000 in 5% BSA solution at 4 °C overnight after blocking with 5% BSA for 2 h at room temperature. The plates were washed with PBSt three times for 5 min each time and incubated with HRP-conjugated anti-mouse IgG (CSB-PA644737, Cusabio, USA) at room temperature for two hours. Following 3 washes with PBSt, the plates were incubated with TMB substrate (Komabiotech, Korea) in the dark for 5–10 min, followed by incubation with stop solution (Komabiotech, Korea). Absorbance (optical density) values were measured at a wavelength of 450 nm using a VICTOR^®^ Nivo^™^ Multimode Plate Reader (PerkinElmer).

### 2.9. Cell Fractionation and Surface-Expressed MHV Spike Protein Detection

Monolayers of HEK 293T cells in 100 mm dishes were transfected with 13 µg each of pCAGGS cl-2 S and pCAGGS JHM-X S, and subsequently, the plasma membrane fraction proteins were isolated following the protocol for a Minute Plasma Membrane Protein Isolation and Cell Fractionation Kit (Invent Biotechnologies, Inc., Plymouth, MN, USA). Five micrograms of each cytosolic and plasma membrane fraction were separated in a 10% SDS gel and transferred to a PVDF membrane. Each fraction was confirmed using marker antibodies against pancadherin (#4068, Cell Signaling, Danvers, MA, USA) for the plasma membrane fraction and β-actin (SC-47778, Santa Cruz Biotechnology, Dalla, TX, USA) for the cytosolic fraction; subsequently, the spike proteins were detected with MHV spike antibody (MHV 10 G).

### 2.10. Cell Fusion Assay

Fusion assays were performed using two cell lines: DBT and 293T. HEK 293T cells were seeded in 12-well cell culture plates and transfected with the same amount of pCAGGS, pCAGGS cl-2 S, or pCAGGS JHM-X S plasmid DNA. Forty-eight hours after transfection, the cells were harvested, and 5 × 10^4^ cells in DMEM, 10% FBS were overlaid on DBT cells at 80–90% confluence following incubated in a CO_2_ incubator at 37 °C for 12–15 h. Fusion photography and area quantification were performed using the OptiView camera program (Korea Lab Tech, Seongnam, South Korea).

### 2.11. Quantification of Cell Fusion by Luciferase Activity

A luciferase fusion assay was performed using the dual split plasmids DSP_1–7_ and DSP_8–11_ in a pIRES puro3 vector mentioned in previous studies [27,28]. Monolayers of DBT and 293T cells were prepared in 12-well cell culture plates, and DBT cells were transfected with DSP_8–11_ plasmid or DSP_8–11_ plasmid and DSP_1–7_ plasmid as the control. In addition, 293T cells were transfected with both DSP_1–7_ plasmid and cl-2 spike or JHM-X spike plasmid. Twenty-four hours after transfection, 293T cells expressing DSP_1–7_ and MHV spike protein were harvested and overlaid on DBT cells expressing DSP_8–11_. At the time specific CPEs appeared (12–16 h post transfection), the cells were harvested, and the cell pellet was mixed with EnduRen Live cell substrate (E6481, Promega, USA) diluted in PBS as described in the manufacturer’s protocol. Then, 100 µL of the mixture was added to each well of a white cell culture plate (SPL Korea). Cells were incubated for 1–2 h in a CO_2_ incubator at 37 °C, and subsequently, luciferase activity was measured using a VICTOR^®^ Nivo^™^ Multimode Plate Reader (PerkinElmer). To express the luciferase activity relative to the amount of spike protein expressed, the spike protein expression level was estimated quantitatively via ELISA and western blotting at the time of overlaying. ImageJ software was then used to measure the intensity of the western blot bands. Next, the relative luciferase units (RLU) obtained for JHM-X were divided separately using the extraneous fold values obtained for the JHM-X spike protein amount using an ELISA and western blotting.

### 2.12. Statistical Analysis

All statistical analyses were performed using GraphPad Prism 8 software. We expressed our results as the mean ± standard deviation (SD) and compared the mean values using the Student’s *t*-test.

## 3. Results

### 3.1. Virus Growth Comparison and Sequence Difference in the HVR of cl-2 and JHM-X

We compared the growth patterns of the two viruses at different time points. As shown in Figure 1A, JHM-X started to grow at 6 h post inoculation (hpi) and reached PFUs of 3.3 × 10^6^ (12 hpi), 9.6 × 10^6^ (18 hpi), 1.2 × 10^7^ (24 hpi), and 7.2 × 10^6^ (30 hpi). Cl-2 also started to grow at 6 hpi and reached PFUs of 1.4 × 10^5^ (12 hpi), 2.3 × 10^5^ (18 hpi), 1.57 × 10^5^ (24 h hpi), and 5.0 × 10^4^ (30 hpi). We found an approximately 100-fold difference in the titer at 18 hpi. To confirm the potential reason for this growth difference, we compared previously reported spike gene sequences for both viruses and found that JHM-X had a large deletion (153 amino acids) in the spike protein, especially in the HVR (Figure 1B). Comparison of isolated spike gene sequences from viral RNA also confirmed that the JHM-X spike protein retains the same amino acid deletion (Supplementary Figure S1), as reported previously [6]. However, it is not clear whether this deleted region is involved in the virus growth difference or not and it needs a deep study of the genome and the function of both viruses. Since viruses grew very fast at higher MOI, viruses reached their peak titers within a short time. At lower MOI (0.0002), viral copy numbers of each virus at 6, 12, 18, and 24 h post infection were also analyzed to obtain more quantitative evidence for the virus growth difference. Thus, it revealed an almost similar pattern of growth difference between JHM-X and cl-2 compared to the growth difference observed in plaque assay (Figure 1D). Altogether it was revealed that those two viruses have significantly different growth rates in DBT cells although the mechanism has yet to be elucidated.

### 3.2. Plaque Size Comparison between the Two Viruses in DBT and BHKR1 Cells

**Figure 2:**
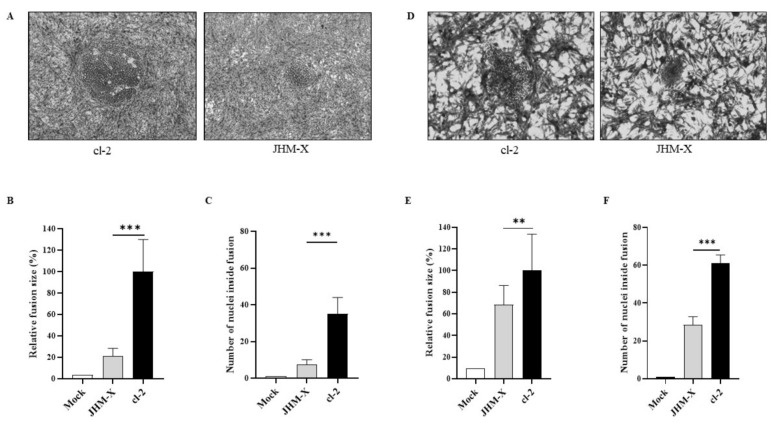
Plaques formation and fusion size difference between cl-2 and JHM-X in DBT and BHKR1 cells. (**A**) Syncytia produced by cl-2 and JHM-X in DBT cells. DBT cells prepared in 24-well plates were infected with MHV cl-2 or JHM-X (MOI = 0.01) and observed under a microscope 15 h post infection. (**B**) The area of more than 20 plaques consisting of fused cells was analyzed using eXpo ver. 5.0.1 microscopic imaging software, and the average area and standard deviation were calculated. The average plaque sizes of JHM-X and cl-2 were 14,010.7 μm^2^ and 25,468.3 μm^2^, respectively. Data are presented as the percentage of the mean fusion area of cl-2. (**C**) DBT cells prepared in 24-well plates were infected with MHV cl-2 or JHM-X (MOI = 0.01) and observed under a microscope 9 h post infection, and the number of nuclei inside more than 10 plaques was counted. (**D**) BHK cells prepared in 24-well plates were infected with MHV cl-2 or JHM-X (MOI = 0.01) and observed under a microscope 15 h post infection. (**E**) The area of more than 20 plaques consisting of fused cells was analyzed, and the relative fusion area was calculated by dividing all fusion areas by the mean fusion area of cl-2. (**F**) The number of nuclei inside a plaque was counted at a time similar to that in DBT cells. The data were analyzed using GraphPad Prism 8 software. The results revealed that the number of nuclei inside a plaque was significantly different. The vertical line extending above each column indicates the standard deviation; ** and *** denote statistically significant differences (*p* < 0.001 and *p* < 0.0001, respectively, determined by a Student’s *t*-test).

We were curious about the underlying cause of the growth changes. To answer this question, we first compared each plaque size and the number of cells involved in each plaque. As shown in Figure 2A, cl-2-infected cells generated much larger plaques than JHM-X-infected cells. We found that the biggest difference occurred at 15 hpi.

We randomly selected 30 plaques and measured the size of each plaque, and the plaque area was presented as a percentage of the total area of the image. The mean percentage obtained for JHM-X was 0.8141 and for cl-2 was 3.5490, showing a significant difference (Figure 2B). Additionally, there was a significant difference between the two strains in the cell numbers involved in each plaque. As shown in Figure 2C, there were approximately 10 JHM-X cells and 43 cl-2 cells. In conclusion, we found that the size of each plaque and the number of cells involved in each plaque were significantly higher in cl-2-infected DBT cells than in JHM-X-infected DBT cells.

To confirm that the results were not specific only to DBT cells, we performed the same experiments in BHK cells. To compare fusion activities between JHM-X and cl2, we compared plaque size and the number of cells involved in each plaque for BHKR1 cells expressing the MHV receptor CECAM1a. As shown in Figure 2D, cl-2-infected BHKR1 cells generated much larger plaques than JHM-X-infected cells. We found that the largest difference occurred at 15 hpi, similar to DBT cells.

We randomly selected 30 plaques and measured the size of each plaque. The mean percentages of plaque sizes were 1.2929 for JHM-X and 1.8275 for cl-2 showing a significant difference, similar to DBT cells (Figure 2E). Moreover, the number of cells involved in each plaque was also significantly different between the two strains (Figure 2F). We found that the size of each plaque was significantly greater in cl-2-infected BHKR1 cells than in JHM-X-infected BHKR1 cells. Hence, BHKR1 cells showed a similar pattern of plaque size differences after viral infection, supporting the notion that cl-2 can induce comparably larger plaques than JHM-X. Thus, we confirmed that the plaque size differences and the number of cells involved in each plaque are not cell dependent but caused by native activities.

### 3.3. Fusion Formation Comparison of Spike Proteins Expressed by the Two Strains

**Figure 3:**
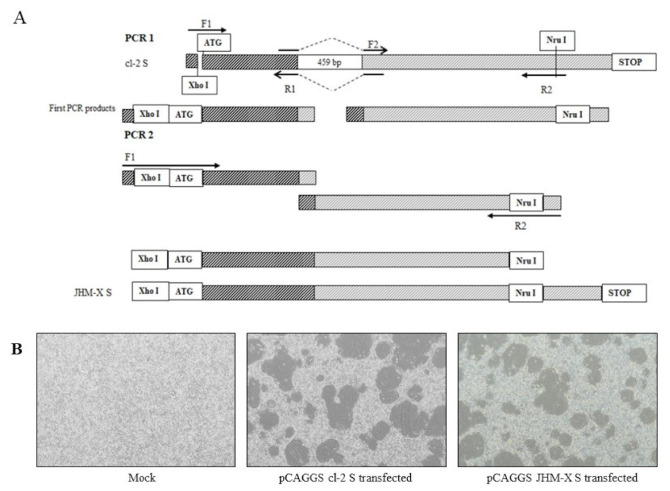
Recombinant spike protein generation and CPE formation in transfected DBT cells. (**A**) The upstream region was amplified using a forward primer (F1) with an existing restriction enzyme site (Xho I) and a reverse primer (R1) with an overhang region overlapping with the downstream region next to the deleted section. Correspondingly, the downstream region was amplified using a forward primer (F2) with an overhang region overlapping with the upstream region above the deleted section and a reverse primer (R2) with an existing restriction enzyme site (Nru I) in the plasmid. The purified PCR product from the 1st PCR was subsequently used as the template for PCR 2 with F1 and R2 primers, and the PCR product from PCR 2 was inserted into a pCAGGS vector using the enzyme sites Xho I and Nru I. (**B**) Recombinant spike protein can generate syncytia in DBT cells, and the fusion difference between the two viruses was not differentiated.

For further study, we compared the results from viruses and expressed spike proteins. A detailed schematic diagram for cloning is presented in Figure 3A. Approximately 15–20 h after transfection, a typical cytopathic effect was shown, and cells were fused with neighboring cells to form syncytia (Figure 3B). Certainly, the syncytia formation of transfected DBT cells was similar to that of virus-infected cells, although the size of fusions and the number of cells inside fusions were not significantly different from the transfected spikes (data not shown).

### 3.4. Quantification of Spike Protein Expression by Two Viruses

**Figure 4:**
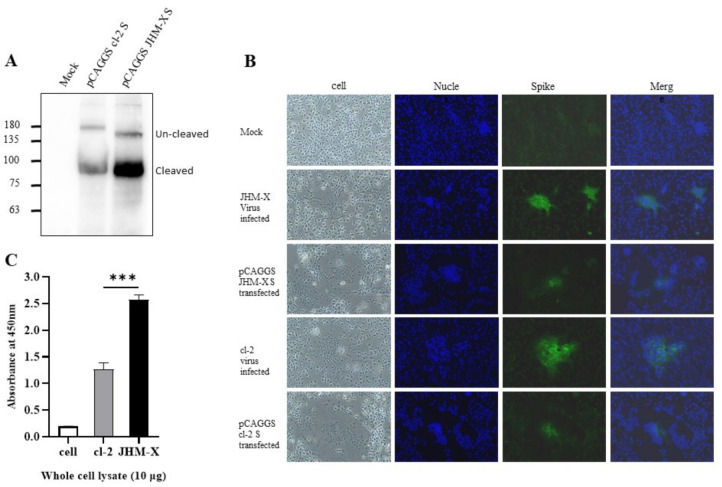
Expression of recombinant spike proteins. (**A**) Expression of MHV spike plasmids in 293T cells was assessed via SDS-polyacrylamide gel electrophoresis using MHV spike-specific antibody 10 G. (**B**). Immunofluorescence imaging revealed that the fusions produced in DBT cells are absolutely caused by the spike protein, and most of the protein is expressed on the cell surface. (**C**) Whole-cell lysates were prepared from mock-, cl-2- and JHM-X-infected DBT cells at the time of specific CPE formation, and the amount of spike protein was quantified by an indirect ELISA using the MHV spike monoclonal antibody 10G. The data were analyzed using GraphPad Prism 8 software. The vertical line extending above each column indicates the standard deviation, and *** denotes a statistically significant difference (*p* < 0.0001 determined by a Student’s *t*-test).

We confirmed expression using western blotting, as shown in Figure 4A. There was clear expression and two forms, cleaved and uncleaved spike proteins. Furthermore, through immunofluorescence using an antibody specific for MHV spike proteins, we detected the fusions produced by transfection with spike proteins (Figure 4B). The size of the plaques and the number of cells involved in each plaque were much larger in virus-infected cells than in virus-transfected cells. These results were the same in both strains. All the spike proteins were expressed only in the cell cytoplasm. Based on DAPI staining, there were no merged cells (Figure 4B).

We were still curious as to why JHM-X growth is approximately 100-fold higher than cl-2 growth. Thus, we speculated that JHM-X produced larger amounts of S protein than cl-2, so the fusion activity of JHM-X was stronger than that of cl-2 because the amount of spike protein expressed was larger. Nevertheless, the difference found in the present study was that cl-2 produced greater fusions than JHM-X (1.5–2-fold). The amount of S protein in infected cells was quantified using virus-infected cell lysates. Interestingly, we found that JHM-X infection produced significantly higher amounts of S protein, while cl-2 infection produced 1.5- to 2-fold lower amounts of the spike protein. (Figure 4C). This was entirely different from our hypothesis. In conclusion, we found that the spike protein on cl-2 was expressed at much lower levels than that on JHM-X, but its fusion activity was much higher than that of the JHM-X spike protein. These results can also be seen in Figure 4A. Even after western blotting, we loaded the same amount on the gel, and the density of spike protein was much larger in JHM-X, indicating a higher protein amount.

### 3.5. Cell Surface Expression of Spike Proteins from the Two Strains

**Figure 5:**
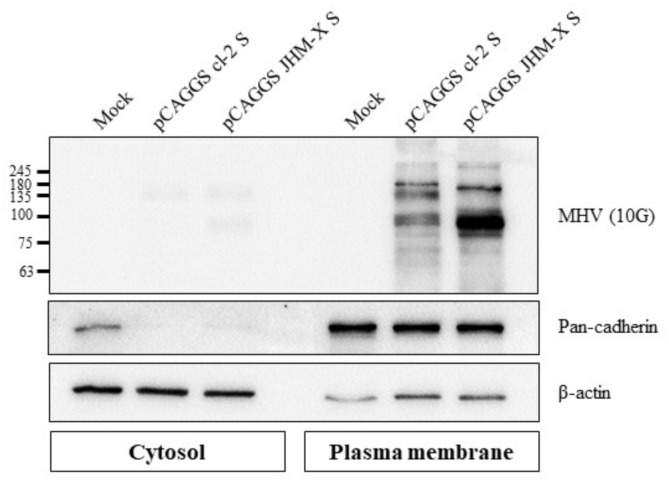
Cell surface expression of the MHV spike protein. Western blot analysis revealed that both MHV spike proteins were expressed on the cell surface, and JHM-X expressed greater spike protein amounts than cl-2.

Subsequently, we successfully isolated the plasma membrane fraction from cells, and cell fractionation was confirmed using fraction markers: pan cadherin for the plasma membrane fraction and beta actin for the cytosolic fraction (Figure 5). The spike protein expression level in the plasma membrane fraction was greater than expression in the cytosol, and these results indicated that the higher expression level of JHM-X spike protein than cl-2 spike protein was consistent with the previous results obtained with ELISAs.

### 3.6. Comparison of Cell Fusion Activity

**Figure 6:**
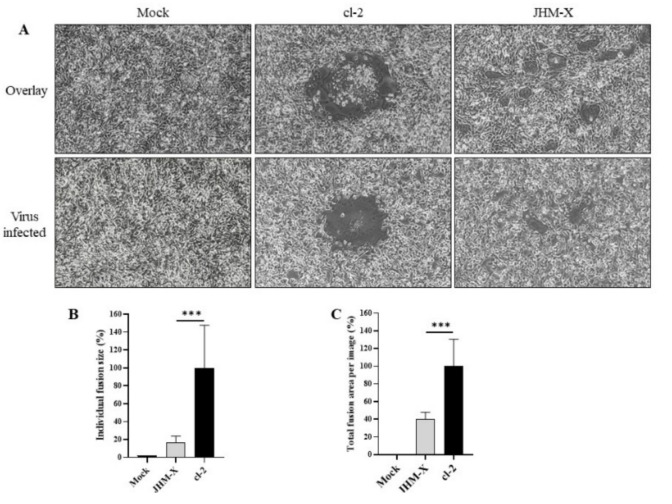
Cell fusion assay. (**A**) Forty-eight hours after transfection, 293T cells were harvested and overlaid on DBT cells or DBT cells were infected with viruses as the positive control, and 12–15 h later, fusion formation was induced. (**C**) The area of 40 individual fusions was calculated, and the area of all fusions was divided by the mean area of cl-2 fusions to obtain the relative fusion size. (**B**) The average total fusion area per image based on 20 images was calculated and is presented as a percentage of the cl-2 fusion area. The vertical line extending above each column indicates the standard deviation, and *** denotes a statistically significant difference (*p* < 0.0001 determined by a Student’s *t*-test).

Next, we compared the fusion activity of spike proteins from both viruses to understand how cell fusion activities are different. Transfection of 293T cells with either cl-2 or JHM-X spike protein induced the formation of fusions with DBT cells (Figure 6A). Furthermore, careful observation of the fusions formed by two spike-expressing cells revealed a similar pattern of fusion difference; cl-2 exhibited higher fusion activities than JHM-X. We also compared the virus-infected strains (Figure 6A). To express the data quantitatively, the fusion area from 40 individual fusions was calculated, and each area is presented as a percentage of the mean fusion area of cl-2, emerging 17.16% and 100% mean fusion area, respectively, from JHM-X and cl-2. Statistical analysis revealed a significant difference in the fusion activity after spike expression (Figure 6B). To further confirm the fusion size difference, the sum of the area of the total fusions inside an image was calculated from 20 images and similarly presented as a percentage of the mean fusion area of cl-2. The mean percentage for JHM-x was 7.65, while cl-2 represented 100% fusion activity (Figure 6C). Altogether, these data show the ability of the cl-2 spike protein to form greater fusions than the JHM-X spike protein and the contribution of the deleted region to the strong fusion activity.

### 3.7. Fusion Size Quantification Using Luciferase Activity

**Figure 7:**
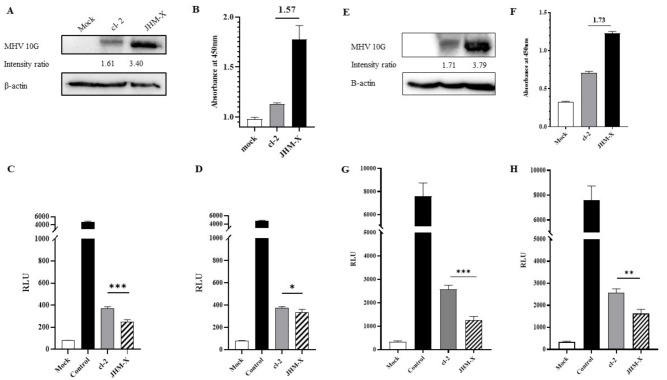
Quantification of cell fusion by luciferase activity. (**A**) Mock-, cl-2-, and JHM-X-transfected cells were harvested, and western blotting was performed to assess the amount of spike protein expressed by both spike plasmids at the time of cell overlay on DBT cells using MHV 10G antibody and β-actin antibody, and the band intensity was measured using ImageJ software. (**B**) At the same time, the amount of spike protein was measured quantitatively via ELISA, and the difference between absorbance values is indicated on the graph. (**C**) DSP8–11-expressing DBT cells were subsequently overlaid by 293T cells co-transfected with DSP1–7 and cl-2 spike protein or JHM-X spike protein. Twelve to 15 h after overlaying, RLUs were measured. The RLUs obtained for JHM-X in DBT cells were divided by the additional fold value of spike protein amount expressed by JHM-X depending on the western blot band intensity ratio. (**D**) RLUs obtained for JHM-X in DBT cells were divided by the additional fold value of spike protein expressed by JHM-X depending on the ELISA results. Similarly, a luciferase fusion assay was performed on BHKR1 cells. (**E**) According to the western blotting, spike protein expression level at the time of overlaying on BHKR1 cells. (**F**) Spike protein amount quantification using ELISA at the time of overlaying on BHKR1 cells. (**G**) The RLUs obtained for JHM-X in BHKR1 cells were divided by the additional fold value of spike protein amount expressed by JHM-X depending on the western blot band intensity ratio. (**H**) RLUs obtained for JHM-X in BHKR1 cells were divided by the additional fold value of spike protein expressed by JHM-X depending on the ELISA results. The vertical line extending above each column indicates the standard deviation, and *, ** and *** denote statistically significant differences (*p* < 0.05 and *p* < 0.001, *p* < 0.001, respectively, determined by Student’s *t*-test).

To further confirm the fusion activity difference between the two strains, a luciferase assay was performed using dual split proteins (DSPs). Both western blotting and ELISA results confirmed that the spike protein expression level of JHM-X was much higher than that of cl-2 (Figure 7A,B). According to the band intensity ratio in western blots, JHM-X expressed 2.11-fold higher spike protein than cl-2, while ELISA results indicated 1.57-fold higher spike protein production. Therefore, we divided the RLUs of JHM-X by the extraneous spike protein amount of JHM-X to obtain the absolute fusion size difference between cl-2 and JHM-X. As shown in Figure 7C,D, we found that the fusion activities were significantly different. We performed the same experiment in BHKR1 cells to give strong evidence for the different fusion activities of the two viruses. At the time of overlaying we could detect greater spike protein expression level by western blot and ELISA. (Figure 7E,F). When comparing the western blotting band intensity ratios of cl-2 and JHM-X, JHM-X expressed 2.21 fold more spike protein amount than cl-2 (Figure 7E) and we divided the RLU value obtained for JHM-X by 2.21 followed by graphed as shown in Figure 7G. By ELISA, it was detected 1.73 fold more spike protein amount from JHM-X (Figure 7F) and RLU value obtained for JHM-X was divided by 1.73 to obtain the absolute fusion size difference following graphed in Figure 7H. In conclusion, the expression level of the spike protein is involved in the formation of cell-cell fusion subsequently, cl-2 produced significantly strong fusion activity than JHM-X under similar expression levels of the spike protein.

## 4. Discussion

In coronaviruses, the spike glycoprotein is the major protein involved in the binding of the virus to cellular receptors for membrane fusion, assisting viral entry into cells. Essentially, the S1 subunit is important for attachment to the cellular receptor, while the S2 subunit is involved in membrane fusion. Subsequently, the S protein translocates to the cell surface and induces the formation of syncytia with neighboring cells [10,11,17,29]. Most of the disparities in the host range and the tissue tropism between coronaviruses are characterized by alterations in the spike glycoprotein. Furthermore, the spike protein is the major pathogenic determinant of coronaviruses, and the diversity of virulence is mostly due to differences in the spike gene. Substitution of the spike gene in an attenuated viral strain genome with the spike gene of a highly virulent strain allows virus entry into particular cells and enhanced pathogenesis [13,29]. Previously, it was reported that pseudorecombinant viruses; expressing the spike proteins from both enteric and respiratory transmissible viruses could be found in the enteric tract, while helper virus had the respiratory tropism, realizing the ability of the spike protein to determine tissue tropism and virulence [30]. MHV4 is a highly neurovirulent strain, and replacing the spike gene of MHV4 with that of the mild neurovirulent strain MHV-A59 via targeted RNA recombination led to high neurovirulence of a mildly neurovirulent strain, indicating the importance of the MHV spike protein in pathogenesis [31]. Moreover, previously, fusion-defective mutants of MHVs were found to be attenuated and to display diverse pathogenic properties [32]. Overall, the spike protein contribution to coronavirus pathogenesis is significant.

As previously reported, several studies have shown the presence of MHV variants with deletions in the spike protein. These deletions were discovered in the HVR of the S1 subunit or near the heptad repeat regions of the S2 subunit, and the nature and significance of the deletions remain unclear [5,31]. However, previous studies have investigated whether deletions or mutations in this region can alter viral fusion, receptor binding, and viral spread or eliminate neutralizing monoclonal antibody epitopes and abrogate CD8+ T-cell epitopes. The deletion variant of MHV4, V5A13.1, consisting of the 142 amino acid deletion in the HVR in the S1 subunit, was neuroattenuated in mice, confirming the significance of the deletion region in pathogenesis [5,11,31].

Our study provides further information on the biological significance of the 153 amino acids that exist in cl-2 but not in JHM-X. The titer of JHM-X was approximately 100-fold higher than that of cl-2 (Figure 1A), but the size of syncytia formed in infected cells was higher after infection with cl-2 than JHM-X (Figure 2A,D). Since the spike protein of coronaviruses is involved in cellular attachment, entry and cell–cell or virus fusion, we focused our study on determining the cellular fusion differences. We found that the fusion size of cl-2 was significantly higher than that of JHM-X in DBT and BHKR1 cells (Figure 2). Based on all these results, we confirmed that the fusion sizes were different.

Studies on coronavirus spike proteins, including the SARS-CoV-2 spike protein, have revealed the cell surface expression of the recombinant spike protein in different cell lines. Spike protein of infected cells and S-transfected cells transported to the cell surface subsequently mediates fusion with neighboring cells following the formation of syncytia [17,33,34,35,36]. To assess the surface expression of the MHV spike, the plasma membrane fraction of MHV spike-transfected cells was isolated and subjected to western blot analysis. The results revealed significantly higher surface expression with JHM-X than cl-2; thus, based on the results presented in Figure 5, we concluded that not only the amount of expression in the cells was higher but also the localization was mostly on the cell surface. Cell surface expression of spike protein reflects the ability of the spike protein to attach to the receptors of neighboring cells and form cell-to-cell fusions. Thus, we performed an overlay assay for fusion activity comparison. We found that cl-2 spike protein generated larger fusions than the JHM-X spike protein, even though the level of JHM-X spike protein expression was much higher than that of cl-2 spike protein.

Studies on fusion-defective mutants and mutations in the spike protein of MHV have explored whether inadequate cleavage of the MHV spike protein due to the deduced amino acid sequence in the S1–S2 junction resulted in small plaques and a low level of cell-to-cell fusion in fibroblasts, whereas wild-type MHV A59 showed complete cell fusion. In addition, the mutant-infected cells produced complete plaques, similar to the wild-type virus, at the delayed time points of infection [37]. In our study, we found a fusion difference 15–16 h after virus infection, and the plaque size of JHM-X approached a size similar to that of cl-2 at the later time points, similar to a previous study (data not shown). When comparing the spike gene sequences of JHM-X with those of cl-2, the 153 amino acid deletion in the HVR of the S1 subunit is the only defective region in JHM-X [6]. Therefore, we speculate that the deletion in the HVR of JHM-X could be involved in conformational modifications in the spike protein and consequent defects in cleavage, subsequently leading to the formation of a small plaque phenotype in cultured cells. Further studies are essential to confirm the mechanism behind this phenomenon.

In the same study on MHV A59 and its mutants, it was shown that the viral growth kinetics of both viruses were similar, although the plaque sizes were different [37]. Likewise, a separate study demonstrated that variant strains with deletions in the HVR of MHV-4 can augment the growth of cultured cells relative to the wild-type virus MHV-4 [38]. Equivalently, our findings show that JHM-X, which has a deletion in a similar region, has a 100-fold higher viral titer than the wild-type strain cl-2, indicating that the region deleted in the S1 subunit is superfluous for virus growth in vitro. However, this finding was not consistent in all cell cultures [36].

We still do not know the underlying cause of all these results. We confirmed that the 153 amino acid region in the HVR of the spike protein is important for strong fusion activity, although the mechanism underlying the difference in fusion formation is not clear.

## 5. Conclusions

In conclusion, our study confirmed that deletion of the HVR region of the spike protein of MHV caused remarkable differences in fusion activities. This might be related to its pathogenesis, but further studies are needed to confirm the mechanism and the other cellular factors involved in this phenomenon.

## Figures and Tables

**Table 1:** Primers used for the construction of pCAGGS JHM cl-2 S.

Primer	Nucleotide Sequences
Forward 1 (F1)	5′ TACCCGGGCATGCTCGAGATGCTGTTCGTCTTTATTTTACTATT 3′
Forward 2 (F2)	5′ GTTACCATAAATAACTATAAAAATGATCACTCACAAATTT 3′
Reverse 1 (R1)	5′ AAATTTGTGAGTGATCATTTTTATAGTTATTTATGGTAAC 3′
Reverse 1 (R2)	5′ TCGCGAACTTCTTGACCACCAGTGCAATTG 3′

## Data Availability

Data supporting the reported results can be requested from the corresponding author.

## Supplementary Materials

The following supporting information can be downloaded at: https://www.mdpi.com/article/10.3390/v14020398/s1, Figure S1: Amino acid sequence alignment of cl-2 and JHM-X spikes.
