# Supplementary material for: Reduction of Cell Fusion by Deletion in the Hypervariable Region of the Spike Protein of Mouse Hepatitis Virus
Source: Viruses. 2022 Feb 15;14(2):398. doi: 10.3390/v14020398 (PMC8876987; doi:10.3390/v14020398)
Supplement: Supplementary file 1 [file viruses-14-00398-s001.zip › viruses-1517547-supplementary.pdf]

viruses

MDPI

Supplementary Material

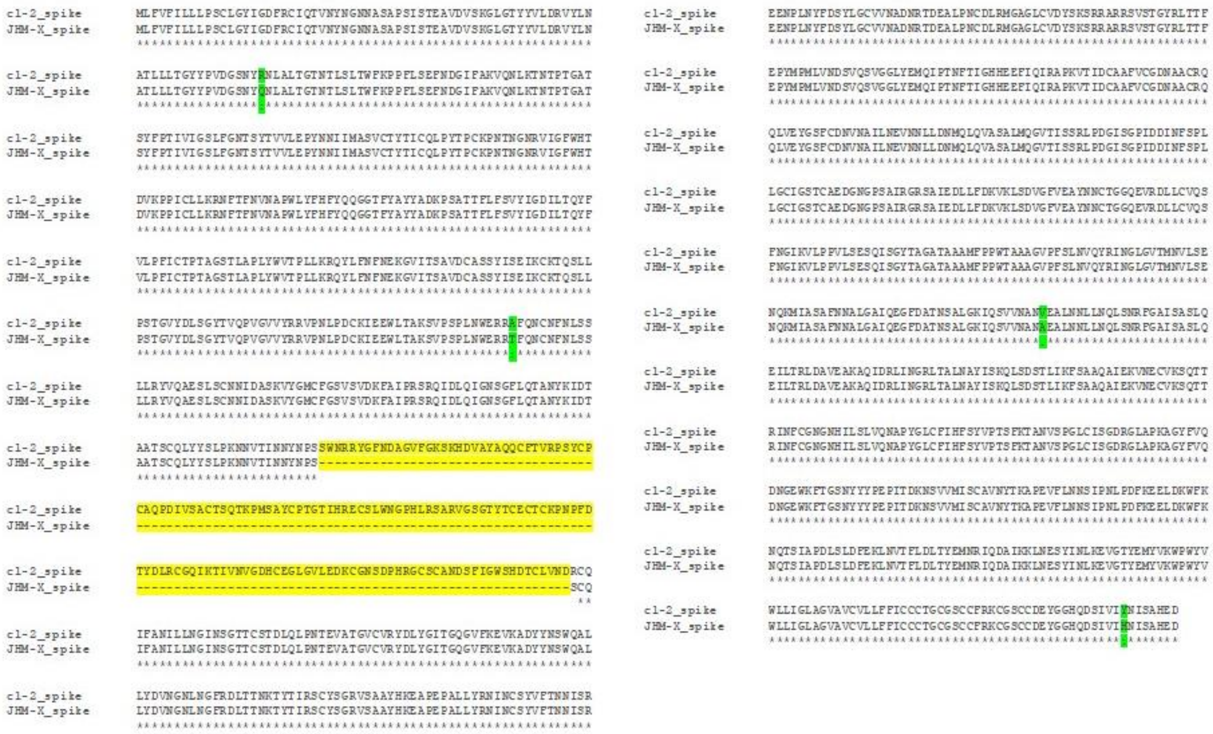

Figure S1. Amino acid sequence alignment of cl-2 and JHM-X spike proteins. The amino acids marked in yellow indicate the 153 amino acid deletion in the HVR of the JHM-X spike protein, and green indicates other mutations.
